# Variation of Basal EROD Activities in Ten Passerine Bird Species – Relationships with Diet and Migration Status

**DOI:** 10.1371/journal.pone.0033926

**Published:** 2012-03-29

**Authors:** Miia J. Rainio, Mirella Kanerva, Niklas Wahlberg, Mikko Nikinmaa, Tapio Eeva

**Affiliations:** 1 Section of Ecology, Department of Biology, University of Turku, Turku, Finland; 2 Division of Genetics and Physiology, Department of Biology, University of Turku, Turku, Finland; Pennsylvania State University, United States of America

## Abstract

Inter-specific differences in animal defence mechanisms against toxic substances are currently poorly understood. The ethoxyresorufin-*O*-deethylase (EROD) enzyme plays an important role in defence against toxic chemicals in a wide variety of animals, and it is an important biomarker for environmental contamination. We compared basal hepatic EROD activity levels among ten passerine species to see if there is inter-specific variation in enzyme activity, especially in relation to their diet and migration status. Migratory insectivores showed higher EROD activity compared to granivores. We hypothesize that the variable invertebrate diet of migratory insectivores contains a wider range of natural toxins than the narrower diet of granivores. This may have affected the evolution of mixed function oxidases (MFO) system and enzyme activities. We further tested whether metabolic rates or relative liver size were associated with the variation in detoxification capacity. We found no association between EROD activity and relative (per mass unit) basal metabolic rate (BMR). Instead, EROD activity and relative liver mass (% of body mass) correlated positively, suggesting that a proportionally large liver also functions efficiently. Our results suggest that granivores and non-migratory birds may be more vulnerable to environmental contaminants than insectivores and migratory birds. The diet and migration status, however, are phylogenetically strongly connected to each other, and their roles cannot be fully separated in our analysis with only ten passerine species.

## Introduction

Birds, as other animals, have molecular mechanisms for detoxifying harmful compounds, including contaminants [1]. Birds are able to modulate their enzyme activities and detoxification systems as a response to pollution levels and in that way improve their survival under pollution exposure in polluted areas [2]. However, inter-specific differences in bird defence mechanisms against toxic substances are poorly understood. Species specific differences in detoxification capacity are suggested to be diet-bound [3], [4]. For example, species having normally high levels of harmful secondary compounds in their diet or species using toxic food items might also tolerate higher levels of contaminants and have better ability to detoxify harmful compounds than species whose diet naturally contains low amounts of toxic chemicals [5]. Furthermore, different feeding habits ranging from omnivorous species to specialists (e.g. insectivores and granivores) can be related to inter-specific differences in detoxification capacity. Omnivores, which use a mixture of plant and animal matter as their food, show high detoxification capacity whereas species with narrow diet may show lower detoxification capacity [3], [4]. Earlier reviews by Ronis & Walker [6] and Sinclair & Sinclair [7] have reported a strong relationship between hepatic microsomal mono-oxygenase (MO) activities and diet, based on studies on 30 species of birds in 10 different orders. There was also an inverse relationship between body weight and relative MO activity in fish-eating birds and mammals [6], [7], suggesting that there may be a relationship between MO activity and metabolic rates that are size-dependent. It is therefore possible that e.g. insectivorous and granivorous birds differ from each other in their detoxification capacity due to their different diet composition.

Migratory behaviour may also be linked to detoxification capacity of a species. Migratory birds, especially those moving between boreal and tropical environments, can be exposed to a wide range of toxic substances (e.g. secondary compounds or contaminants) throughout their annual cycle. Resident birds, on the other hand, may face a narrower range of toxic compounds in their relatively small home ranges. Thus, it is possible that birds having a large home range face more diverse toxic compounds in their environment, and are also better adapted to handle variety of harmful compounds, including those of anthropogenic origin. Migratory behaviour and diet are coupled to a large extent. Although migratory insectivores use invertebrate food all year round, they use different insects as food items along their migratory routes and overwintering and breeding areas, which may have led to increased detoxification ability as well as tolerance against the variety of defence chemicals and toxic compounds compared to resident birds.

Species specific differences in detoxification capacity have been found in birds, mammals and fish [3]. The interspecific variation in detoxification capacity is usually related to differences in the mixed function oxidase (MFO) system of animals. The function of MFO enzymes is based on their ability to detoxify compounds into less toxic forms [8]. The members of MFO group are cytochrome P450 enzymes, which are heme-containing proteins that are found in all organisms examined so far [9]. They can metabolize exogenous xenobiotics and endogenous compounds, such as steroids and fatty acids, to a more polar form, which can be more easily excreted from organs [9], [10]. The MFO system that evolved 800 million years ago [11] was probably able to metabolize lipophilic compounds and detoxify natural plant and animal toxins [4]. However, after man-made contaminants appeared in the environment, this enzyme family recognized also the foreign toxic compounds [4], [12]. To a large part the cytochrome P450 group is thought to have evolved in response to natural substances of toxic plants, an idea known as the “plant-animal warfare” hypothesis [12]. In addition, the system metabolizes, e.g. steroid hormones. The many functions of cytochrome P450 enzymes are shown by the large number of genes encoding them. P450 enzymes are mainly concentrated in the liver, where the toxic compounds are modified to more excretable forms by phase I (where MFO enzymes are involved) and phase II biotransformation reactions involving oxidation, reduction, hydrolysis and conjugation [13]. Induction of the P450 enzymes takes place fairly rapidly and elevated activities of P450 can persist for weeks after exposure to contaminants [14]. One of the most important enzymes of cytochrome P450 group, participating in the detoxification process of xenobiotic compounds, is called CYP1A [15]. CYP1A subfamily, which contains the members CYP1A1 and CYP1A2, has been found in all mammals and birds studied so far [16], [17]. CYP1A production increases in cells after chemical exposure and therefore it is a useful marker especially in biomonitoring studies [12]. The basic mechanism in CYP1A induction is the binding of xenobiotics to a cytosolic aryl hydrocarbon receptor (AhR), which initiate the synthesis of proteins such as CYP1A [18], [12]. The enzymatic activity of CYP1A can be assessed by measuring ethoxyresorufin-*O*-deethylase (EROD) activity, which is a sensitive biomarker especially for organic contaminants [12].

EROD activity is a useful biomarker for detecting early signs of contamination [3], [19], [20], [10], [21]. Understanding the innate differences in species-specific detoxification abilities is important for recognizing the species that are at greatest risk in polluted environments [4]. However, when conducting biomarker studies, it is important to keep in mind that species differ in their basal levels, showing species-specific differences even under similar exposure [22]. Therefore the knowledge of basal levels of used biomarkers (e.g. EROD activity) in target species is essential in ecotoxicological studies. Besides enzymatic activity the size of the detoxifying organs, especially the liver, is also an important factor because it may affect the total detoxification capacity and also be related to EROD activity by unit amount of protein. So far, the relation of liver size and specific EROD activity is poorly known in birds.

Basal metabolic rate (BMR), which is the minimum resting metabolic rate in a thermoneutral ambient temperature [23], can be related to EROD activity as well, because metabolic rate affects the production of harmful metabolites. Interspecific variation in BMR has shown to be dependent on many ecological traits such as food, habitat, climate, flight activity, torpor, migration, and latitude and altitude, although phylogenetic analyses have not always supported the adaptivity of such variation [24]. Avian BMR is known to vary especially with body mass, but also other factors including behaviour and environmental conditions can affect the BMR [25]. Daan et al. [26] reported that avian body mass explains 95–97% of interspecific variation in BMR. Birds in general have ca. 30–40% higher BMR than mammals due to their expensive and energy-demanding flight ability [24]. Organ size is often related to BMR as well. Daan et al. [26], for example, showed that birds having relatively high BMR for their body mass had also relatively large kidneys and hearts. Therefore it would be important to include BMR and the size of the organs in the analyses of EROD activity.

Our goal was to find out whether there are differences among passerine species in their ability to detoxify and tolerate harmful compounds in their body. We measured basal hepatic EROD activities in ten passerine species by collecting samples approximately at the same age and time of the year. This was important because EROD activity can have seasonal variation [22]. The standardization of age at sampling is important also to minimize the possible variation caused by different contaminant exposure of wild birds. EROD activities were measured in the absence of known contaminant loads, meaning that contamination loads of the birds are not measured and thus the early contamination in the eggs or later in their nestling phase cannot be completely ruled out, which is often the case with the wild animals caught from their natural habitats. However, because there were no major pollution sources nearby and since all the sampled birds were young with a relatively short period of exposure, we can expect those birds to be relatively uncontaminated and therefore consider their EROD activity as basal activity. The pollution levels in SW of Finland have also shown to be in general the lowest in Europe [27], [28].

So far, the basal EROD activity has been very little studied in small passerine species and our results bring novel information on the levels of EROD activities related to diet and migratory status. To our knowledge this is also the first time that EROD activity is combined with analyses of BMR and relative organ size in small passerine birds. We also took into account the phylogenetic relationships when analysing species-specific variation in EROD activity in relation to relative BMR and relative liver mass. Closely related species are more likely to share the same kind of evolutionary traits such as the MFO system, which has diversified over the course of evolutionary history. We hypothesize that 1) Basal EROD activity varies between different bird species in relation to their diet and/or migratory status; 2) Species ingesting larger amounts of harmful compounds from their natural food are better adapted to them compared to birds ingesting lesser amounts of harmful compounds; 3) Migratory birds have higher detoxification ability than resident birds, due to their more variable diet and living habitats; 4) Insectivores have higher detoxification ability than granivores due to more variable food items containing a variety of different defence chemicals; 5) Birds with higher BMR also show higher EROD activity; 6) High relative liver mass is associated with high EROD activity, both speeding up the detoxification process.

## Materials and Methods

### 1. Ethical statement

The study was conducted in full compliance with Finnish laws and regulation, including the licence of Southwest Finland Regional Environment Centre (permit LOS-2008-L-224-254) for our studies to sacrifice wild birds for scientific purposes. According to instructions of Animal Care & Use Committee of the Turku University, no other licences were needed for this kind of study purposes, when no animal experiments have been done.

### 2. Study species

The study species were great tit (*Parus major*), blue tit (*Cyanistes caeruleus*), chaffinch (*Fringilla coelebs*), greenfinch (*Carduelis chloris*), house sparrow (*Passer domesticus*), willow warbler (*Phylloscopus trochilus*), reed warbler (*Acrocephalus scirpaceus*), segde warbler (*Acrocephalus schoenobaenus*) and reed bunting (*Emberiza schoeniclus*). In addition, we used the data on barn swallows (*Hirundo rustica*), which were collected earlier for other study purposes. All species are common in the area and relatively easy to catch by mist nets. The species were chosen so that they would represent different migratory statuses and feeding habits. The birds were divided into four groups based on their migratory behaviour and diet on the basis of published information on each species [29], [30], [31], [32]: 1) migratory insectivores, 2) migratory granivores, 3) non-migratory/partially migratory insectivores and 4) non-migratory/partially migratory granivores (Table 1). However, our division is quite rough because some granivores (e.g. reed bunting) sometimes also use insects as their food items, especially during their breeding season. Likewise some species (great tit, blue tit and greenfinch) classified as non-migratory may show partial migratory behaviour.

**Table 1:** Study species with sample sizes, average body mass, migratory status, diet, average liver mass, relative liver mass (liver mass/body mass %) average BMR (W) and relative BMR (W/g).

Common name	Scientific name	Sample size	Average body mass (g)	Migratory status 1)	Diet 1)	Average liver mass (g) 2)	Relative liver mass (%)	Average BMR (W) 3)	Relative BMR (W/g)
Sedge warbler	*Acrocephalus schoenobaenus*	13	11.4	Migratory	Insectivore	-	-	0.218	0.019
Reed warbler	*Acrocephalus scirpaceus*	14	11.8	Migratory	Insectivore	0.539 (n = 1)	4.552	-	-
Willow warbler	*Phylloscopus trochilus*	19	8.0	Migratory	Insectivore	0.549 (n = 1)	6.845	0.208	0.026
Barn swallow	*Hirundo rustica*	7	21.3	Migratory	Insectivore	0.805 (n = 41)	3.785	0.315	0.015
Great tit	*Parus major*	19	17.8	Non-migratory/partial migrant	Insectivore	0.674 (n = 16)	3.782	0.300	0.017
Blue tit	*Cyanistes caeruleus*	17	11.2	Non-migratory/partial migrant	Insectivore	0.369 (n = 7)	3.283	0.167	0.015
Chaffinch	*Fringilla coelebs*	12	21.5	Migratory	Granivore	0.877 (n = 20)	4.073	0.373	0.017
Reed bunting	*Emberiza schoeniclus*	16	17.6	Migratory	Granivore	0.870 (n = 1)	4.940	0.300	0.017
Greenfinch	*Carduelis chloris*	22	26.9	Non-migratory/partial migrant	Granivore	0.809 (n = 21)	3.010	0.470	0.017
House sparrow	*Passer domesticus*	19	27.8	Non-migratory	Granivore	1.254 (n = 117)	4.508	0.280	0.010

1) Unpublished data by A.P. Møller.

2) McKechnie et al. 2006 and McNab 2009 [36], [24].

3) Cramp 1992, Cramp and Perrins 1993, 1994a, 1994b [29], [30], [31], [32].

Liver masses are based on the data provided by A.P. Møller and the average and relative BMR are based on the studies by McKechnie et al. 2006 and McNab 2009.

### 3. Sample collection and analyses

#### 3.1. Sampling methods

The study was conducted between August and October in 2008 near Turku (60°26′ N, 22°11′ E) in southwest of Finland, where we had three sampling sites. Each species were attempted to collect as quickly as possible to avoid the seasonal and temperature variation. The collection periods for the species were: great tit (12.8.-26.8.), blue tit (6.8.-20.8), chaffinch (12.8.-25.9.), greenfinch (20.8.-17.9.), house sparrow (12.8.-1.9.), willow warbler (6.8.-16.9.), sedge warbler (6.8.-25.8.), reed warbler (6.8.-18.9.), reed bunting (15.9.-15.10) and barn swallows (21.8.-19.9.2007). Most of the species were caught within a month, thus minimizing the temporal variation. In reed warblers all but one was collected till the 10^th^ of September. Those species that were caught over a time period more than one month, however, did not show remarkable variation between catching time and EROD activity.

The habitats included a common alder (*Alnus glutinosa*) dominated grove, a reed bed (*Phragmites*) and garden habitats. There were no major pollution sources near our study areas, although aerial contamination (e.g. via wind or roads) cannot be fully excluded. However, owing to the tendency of young birds to move and mix a lot in autumn (meaning that their origin varies a lot even within a species) we consider it most unlikely that exposure to pollutants would produce a systematic species-related bias in our data. Barn swallows were collected in 2007 near Turku as well. The birds were caught with mist nets (Ecotone). The nets were checked every 30 minutes. All the birds collected in this study were young females (born during summer 2008), 12–22 individuals per species, except in the case of reed warblers and sedge warblers, for which sex determination in field was impossible. The number of these two species was increased to 30 individuals and they were sexed later in the laboratory. By collecting young birds we were able to exclude the possible influence of migration and the diet consumed in their overwintering grounds, as well as reduce variation in their life-time pollution exposure and age-related pollutant accumulation. We also wanted to concentrate on one sex to avoid sex specific variation in their detoxification capacity. The birds were measured for their wing length and body mass. As a condition index we used wing length (mm) relative to body mass (g). Also moulting phase, an index of visible subcutaneous fat and muscle index were recorded and used to define the condition of the birds at time of capture. Birds were sacrificed by decapitation and subsequently dissected for the liver tissue, which was separated and placed in plastic Eppendorf tubes. Liver was chosen as a sampling site, because it is the major site for detoxification process (Whyte et al. 2000). Liver tissue were stored immediately in liquid nitrogen and later kept at −80°C until laboratory analyses. The carcasses of the birds were stored for sex identification and future analyses. The data were collected under the license of the Environmental Centre of southwest of Finland.

#### 3.2. EROD activity analyses

Frozen liver samples were homogenized with TissueLyser (Qiagen, Austin, USA) at +4°C to prevent the loss of enzymatic activity prior to measurement. It is important to keep the samples on ice at all stages of preparation. Centrifugation (10 000 g, 15 min) was conducted at +4°C and the used homogenizing buffer was kept ice-cold as well [33]. The complete supernatant was used in EROD activity analyses. The measurement of EROD activity is based on the function of CYP1A enzyme, which converts 7-ethoxyresorufin to a fluorescent product resorufin, which can be detected with a fluorometer [12]. The amount of resorufin grows linearly as a function of time and to make the samples comparable, the activity was proportioned to total protein concentration. The unit for EROD activity is hence ηmol/min/mg protein. EROD activity was measured according to Burke and Mayer [33] with adaptations to microplate. The measurements were done in triplicates using 96-well plates with Envision microplate reader (Perkin-Elmer Wallac, Turku, Finland). Protein assay was conducted according to Bradford method [34] using BioRad stock diluted to dH_2_O (1∶5) and BSA (1 mg/ml) as a standard and measured with Envision microplate reader at absorbance of 595 nm.

#### 3.3. Statistical analyses

The inter-specific variation in EROD activity was analysed with general linear models (GLMs with Type III SS) in IBM SPSS Statistics software 19.0 [35]. The species was used as an independent variable in the model. Pairwise comparison between the species was done using Tukey's test. The variation in EROD activity between diets and migration status were also analysed with GLMs using diet and migration status as independent variables. EROD activity was log_10_ transformed before the analyses to normalize distributions. Within-species correlations between the variables (EROD, condition index and fat index) were analysed using Spearman's correlations, because variables were not normally distributed.

We combined the data from basal metabolic rates collected from the literature [36], [24] and the data of liver masses of the studied species (unpublished data of A.P. Møller) to our data to see how EROD activity is related to relative BMR (W/body mass [g]) and relative liver mass (liver mass [g]×100/body mass [g]) (average values for the species) (Table 1). The average liver masses for the species were derived from samples collected between June and September to minimize the seasonal variation in liver masses. The variation between the months was tested with general linear models and since there was no major monthly variation in liver mass, the mean values based on those four months were included in the final analyses (Felsenstein's correlations). The amount of species varied in the analyses, because liver mass was not available for sedge warbler and BMR for reed warbler respectively. The phylogenetically corrected (OLS) regression (with 95% confidence (CI) and predicted intervals (PI) mapped onto the original data space) and correlations between the variables were analysed with Felsenstein's contrasts in Mesquite phylogenetic software (Version 2.74) [37] by using PDTREE module of PDAP after ensuring the normality of the distributions. Felsenstein's correlations (r_F_) are based on phylogenetically independent contrasts (PIC), enabling correlations among the sets of characters for a number of species that are not statistically independent, due to the common ancestor in their phylogenetic history.

Phylogenetic relationships of the studied species were taken from Treplin et al. [38] and branch lengths of the topology were estimated using DNA sequence data from six gene regions. The DNA sequences were downloaded from GenBank (Table S1) and analyzed in a maximum likelihood framework using PAUP* [39]. In some cases a closely related species had to be used as there were no sequences available for species used in this study (Table S1). The topology was fixed and a standard general time reversible model with a gamma correction to allow for rate heterogeneity was applied to the concatenated six gene dataset. To allow the estimation of the length of the basal branches, two outgroups were included (*Corvus* and *Oriolus*). This produced a phylogenetic tree with branch lengths in substitutions per site per time unit, which was input into Mesquite for the independent contrasts analysis after the outgroups were pruned from the tree. Phylogeny was not included in the analyses of diet and migration status, because these variables only had two categories, and especially diet was so strongly dependent on phylogeny (insectivores vs. granivores) that there would be no power in the analysis to detect phylogenically independent variation with this number of species.

## Results

**Figure 1:**
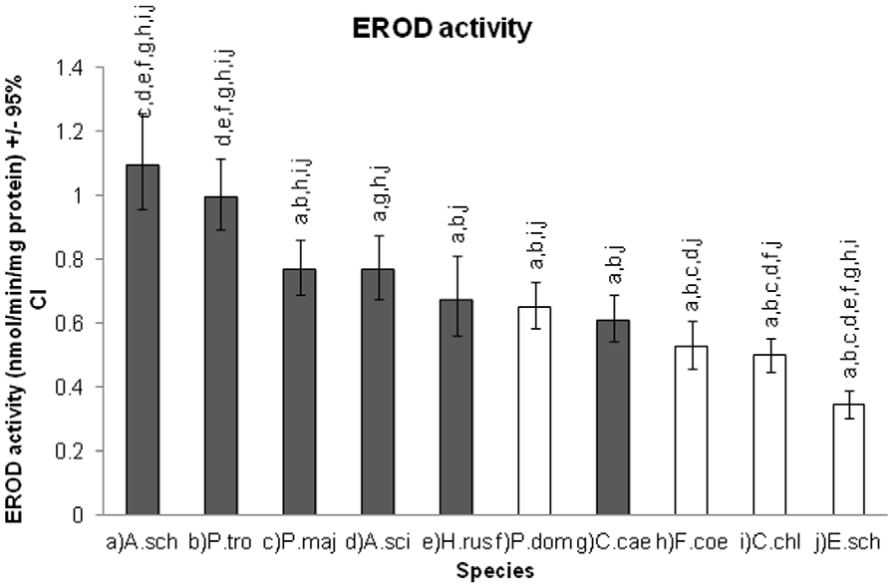
EROD activities (estimated marginal means ±95% CI) in female livers of ten passerine species. Dark grey bars represent insectivores and white bars granivores. Letters above the bars indicate significant differences to the other species (GLM, Tukey's test <0.05): (a) A.sch = *A. schoenobaenus*, b) P.tro = *P. trochilus*, c) A.sci = *A. scirpaceus*, d) P.maj = *P. major*, e) H.rus = *H. rustica*, f) P.dom = *P. domesticus*, g) C.cae = *C. caeruleus*, h) F.coe = *F. coelebs*, i) C.chl = *C. chloris*, j) E.sch = *E. schoeniclus*).

**Figure 2:**
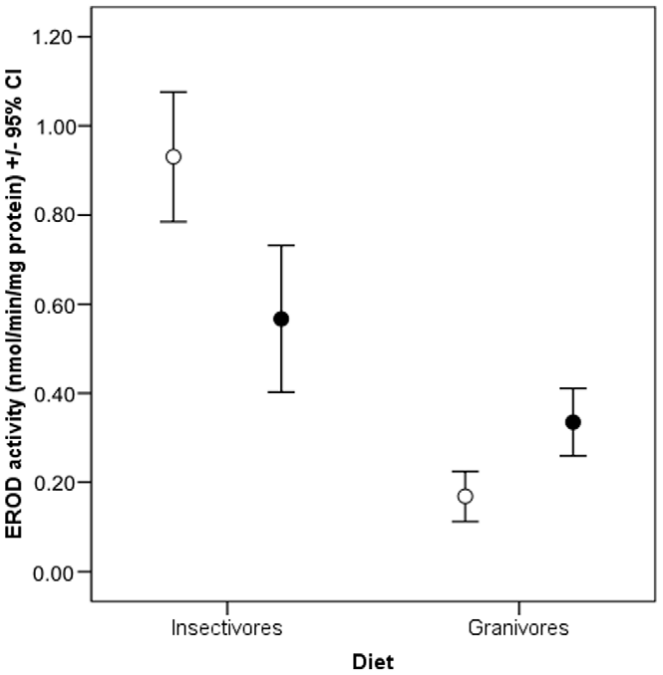
The mean (±95% CI) EROD activity in passerine species with different migratory status and feeding habits. Open circles denote migratory birds and solid circles non-migratory/partial migrants respectively.

The study species differed from each other in their liver EROD activity (GLM: F = 30.2_9_, p = <0.001) (Fig. 1). The EROD activity was significantly associated with the diet (GLM: F = 106.6_1_, p = <0.001) but not directly with the migration status (GLM: F = 0.22_1_, p = 0.64). However, the diet and migration status showed a significant interaction on the EROD activity (GLM: F = 37.6_1_, p = <0.001), indicating highest EROD activity in migratory insectivores (Fig. 2). Migratory granivores showed much lower EROD activity than migratory insectivores (Fig. 2). Non-migratory/partially migrating insectivores showed lower EROD activity levels than migratory insectivores, while non-migratory/partially migrating granivores had higher EROD activity levels than migratory granivores (Fig. 2). However, this result is quite strongly affected by low EROD activity in the migratory reed bunting, which differed significantly from all other study species (Fig. 1). In contrast, the migratory chaffinch had EROD activity levels that were similar to non-migratory granivores (Fig. 1).

There was no significant correlation between EROD activity and condition index or fat index (p-values>0.05) in any other species except willow warbler, which showed negative correlation between EROD activity and fat index (r_S_ = −0.61, p = 0.006, n = 19). This suggests that basal EROD levels are not generally related to the body condition of the birds, though in some species fat reserves may be associated with EROD activity.

**Figure 3:**
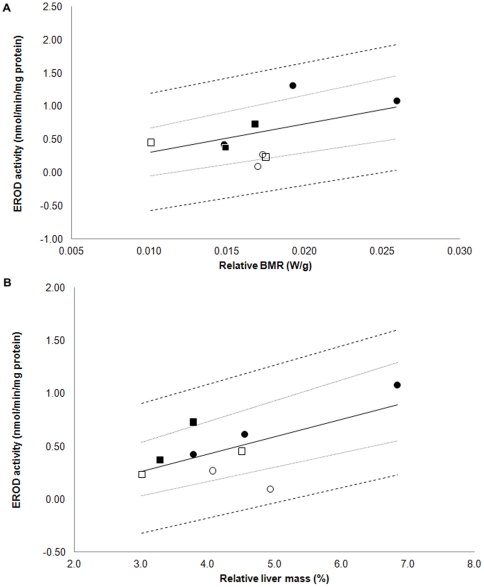
Phylogenetic regressions. Phylogenetically corrected (OLS) regression between EROD activity and relative basal metabolic rate (BMR) (A) and relative liver mass (%) (B) in nine passerine species with associated 95% CI's (dotted lines) and 95% (PI) (dashed lines) mapped onto the original tip data space. Solid circles = migratory insectivores, open circles = migratory granivores, solid squares = non-migratory/partial migratory insectivores, open squares = non-migratory/partial migratory granivores.

After taking account the phylogeny there was no correlation between the body mass (size) of the species and EROD activity (r_F_ = 0.27 p = 0.23, n = 10). Although EROD activity increased with increased relative BMR, the association was not statistically significant (r_F_ = 0.51, p = 0.083, n = 9), (Fig. 3). EROD activity and relative liver mass instead correlated positively with each other (r_F_ = 0.65, p = 0.028, n = 9) (Fig. 3), suggesting that larger relative liver mass is associated with efficient detoxification capacity. Relative liver mass correlated positively also with relative BMR, suggesting that larger relative organ size increases relative BMR (r_F_ = 0.62, p = 0.051, n = 8).

## Discussion

The interspecific variation in basal EROD activity between small passerine species suggests that species differ from each other in their need/capacity of detoxification. Our study indicates that the difference is related to different feeding habits and migratory status, EROD activities being generally higher in insectivorous than in granivorous species. Migratory insectivores showed the highest EROD activity, whereas migratory granivores had lower or similar EROD activity than in non-migratory/partially migratory granivores.

Our results are parallel to the study of Fossi et al. [4], who showed that omnivores, which use variable diet, have more efficient detoxification capacity than specialist birds, which concentrate on only one or a few food items. In our study parids, which use mixed diet (mainly insects in summer and seeds in winter) and could also be considered omnivores rather than pure insectivores, had lower EROD activity than pure insectivores, but still higher than granivores, most probably due to their diet (e.g. moths) containing relatively low amount of defence chemicals. Sometimes also granivores can feed their offspring and themselves with insects, especially during their breeding season making the division between granivores and insectivores less clear. However, the amount and diversity of animal food in granivores is still low compared to pure insectivores and thus the low EROD activity could reflect the relatively low amount of animal food taken in as also the results in parids suggest. Brower [40] and Müller et al. [41] for example have shown insectivorous birds to be able to distinguish prey with low defence chemicals. Also granivores can distinguish the seeds containing high amounts of harmful secondary compounds (e.g. the seeds of the plant families Malvaceae, Convolvulaceae, Rubiaceae and most Leguminosae) and avoid them to get better quality and more digestible food [42]. Fossi et al. [4] had also included one passerine species in their studies, the Italian sparrow (*Passer italiae*), which is mainly a granivore, but feeds also on insects during the breeding season. In agreement with our results, the Italian sparrow had relatively low activities of EROD and aldrin epoxidase, which is another good indicator of detoxification ability. A close relative of Italian sparrow, the house sparrow, also showed relatively low EROD activity in our study, but still higher than in other granivores, which might be due to its more variable diet compared to the other granivores in our study set. However, a direct comparison with the levels in the study of Fossi et al. [4] cannot be done, as they have used liver microsomes, whereas in our study liver homogenate with a different measurement technique was used. Liukkonen-Anttila et al. [43] compared the EROD activities in galliform birds and pigeons and found also species specific variation. Pheasants (*Phasianus colchicus*), which use a variety of different seeds and plant material as food items, showed the highest EROD activity, whereas capercaillies (*Tetrao urogallus*) with their narrow diet showed low EROD activities [43]. Galliformes seem to have relatively low EROD activities compared to Passeriformes examined in our study. However also in the case of Liukkonen-Anttila et al. [43], the measurements have been done using hepatic microsomes and thus the results are not directly comparable. Kennedy et al. [44] have studied the rank order of sensitivity to EROD induction in birds, showing that the chicken (*Gallus domesticus*) was the most sensitive to halogenated aromatic hydrocarbons (HAH), with some other galliform species following the same trend, suggesting that Galliformes in general have relatively low detoxification capacity. In our study, the reed bunting showed very low EROD activity compared to any other species we studied. The reason to this is not known, but it is possible that their main food, such as the seeds of annual weeds (e.g. *Chenopodium*) [45], contains relatively little defence chemicals and therefore their detoxification system may be less efficient. This may be the reason also for the lower level of EROD activity in migratory granivores compared to non-migratory/partial migratory granivores in this study. The chaffinch, with a similar life history to the reed bunting, instead had similar EROD levels with non-migratory/partial migratory granivores.

There are several alternative hypotheses to explain why migratory insectivores showed the most efficient detoxification capacity, as indicated by the highest EROD activities measured. High detoxification capacity in migratory insectivores might be due to their more variable diet containing a wider range of natural toxins that may affect the evolution of their MFO system and further to their ability to tolerate harmful compounds. The lower EROD activity in migratory granivores instead may be related to their narrower and less natural toxins containing diet. Plant species in tropics tend to have more defence chemicals than plants in temperate forests [46]. Tropical forests also have higher variety of plant species, meaning that herbivores and their predators in tropics also encounter greater amount of defence chemicals [47], [48]. Some insect herbivores are able to use those defensive compounds for their own purposes to defend themselves from insectivorous predators [49], [50], [51], [52]. Thus, the large variety of ‘unpalatable’ insects in tropics may have caused birds to develop different detoxification mechanisms to cope with the toxic compounds in their diet [53], [54]. Insectivorous birds for example are the main predators of herbivorous insects during the breeding time of the birds, when also the insect larvae are abundant [55], [56], [57], [58]. Of our study species willow warblers, which showed the highest liver EROD activity, partly overwinter in rainforests [59], but other migratory species inhabit other habitats (e.g. reed beds), having different prey items with different defence chemicals. The pied flycatchers (*Ficedula hypoleuca*), which also overwinter partly in rainforests [59] show high EROD activities as well [10]. Migratory birds also encounter variable diet in their stopover sites during the long distance flights. Since all granivores in our study overwinter in Europe, whereas insectivores in Africa, it would be important to find out whether the overwintering habitat (Europe vs. Africa) explains the different EROD activities between granivorous and insectivorous migrants. The EROD activities between local insectivores and granivores in Africa would also be worth measuring to see whether there are diet related differences in EROD activity, when migration is excluded.

Another hypothesis to explain the variation in detoxification capacity among species is that the energetically expensive long distance migration itself may have produced physiological adaptations between migrants and non-migrants, which can possibly be reflected in their detoxification capacity as well. For example, the relationship between metabolic rate and detoxification capacity is poorly known in birds. It has been shown that BMR is significantly higher in migratory birds than in non-migratory birds [60]. Long distance migrants also often have large intra-annual changes in their BMR, which means that physiological adjustments are associated with different stages of their migratory cycle [61]. In our study set, long-distance migrants had relatively high relative BMR coupled with high detoxification capacity, suggesting that higher metabolic rate could be related to more effective metabolic products. Nonetheless, after correcting for phylogeny the correlation between EROD activity and relative BMR was not so strong.

It is also noted that tropical birds in general have lower BMR than birds living in temperate-zone [62], [63], [64], but see [65], [66]. This is supported also by the study of Wiersma et al. [67], where they found significantly lower BMR in 69 tropical species compared to 59 species living in temperate habitat, even when phylogeny is taken into account. The result did not change either when body-mass-adjusted BMR was used [67]. This is suggested to be due to slower pace of life in tropics, meaning that life-history traits covary with the rate of energy expenditure, tropical birds being at the slow end of the life-history axis and temperate birds at the opposite end [67]. Tropical migrants instead lied there in the middle having higher BMR than birds in tropics but lower than in temperate birds [67], [68]. In our study the relative BMR was highest in small migratory insectivores, whereas the larger migratory granivores had slightly lower relative BMR, followed by non-migrants, suggesting that relative BMR is not only associated with the migration status, but also to the size of the birds. Our result is opposite to the studies of Wiersma et al. [67], though it is possible that the higher relative liver mass in small migratory insectivores affects relative BMR as the positive correlation between them suggests. The elevated mass-specific BMR has been also detected with the larger size of other organs in the study of Daan et al. [26].

The organ masses relative to body mass (e.g. liver, kidney, heart) of the tropical birds have been found to be smaller than those of temperate birds [69]. However, we did not find this kind of trend in our data set, since the trans-Saharan migrants, especially the willow warbler, but also the reed bunting and the reed warbler, had relatively large liver mass in relation to their body mass. The high EROD activity and relatively large liver mass in the willow warbler and the reed warbler might reflect their higher capacity to detoxify secondary compounds as well as pollutants. Despite the large relative liver mass, the reed bunting had very low EROD activity and also other enzyme activities related to glutathione metabolism (Rainio et al. unpublished data), which may be due to its narrow diet. The reed warbler and the sedge warbler, which are close relatives, had very similar level of EROD activities. Also the willow warbler seemed to follow the same trend being also related to other warblers used in this study. The chaffinch and the greenfinch, again close relatives, showed also similar EROD activity levels with each other, although they eat different kind of food. They may still however share the same kind of MFO system. Parid species instead, which are also close relatives differ from each other in their EROD activity, great tits having higher EROD activity than blue tits in relation to their relative liver mass, which may be due to differences in their metabolic machinery since they both use relatively similar food. However, knowledge of the relationship between EROD activity, basal metabolic rate and organ mass relative to body mass in small passerine species is still poor and needs more studies on a larger number of species both at the ecotoxicological and physiological levels to confirm the relationship between those factors.

The diet has been shown to be related to BMR in birds and therefore it may affect species detoxification capacity as well. To our best knowledge this study is the first in small passerine birds, where EROD activity measurements have been combined with BMR and diet. To date, the uric acid levels (indicating protein metabolism) in blood plasma of migratory birds have been shown to be lower in frugivorous species than species feeding mainly on arthropods [70]. Frugivorous birds instead showed high levels of triglycerides, which indicate great dependence on fat metabolism. This suggests that different species use fat and protein at different ratios during migration and species using different diets most probably also accumulate fat and protein as fuel in different ratios [70]. The studies on food habits hypothesis (FHH) have shown contradictory results of the relation of BMR and dietary habits among the species [71], [24], [72], [73]. The hypothesis predicts that species/populations using food with low energy content and/or low digestibility as well as unpredictable food availability will have low mass-independent BMR [71], [74], [75], [76]. McNab [24] has concluded that omnivore species have higher BMR than species feeding only insects, seeds or aquatic invertebrates. Sabat et al. [73] instead found no correlation between the BMR and diet among passerine bird species, probably due to different evolutionary response to natural diets [77]. In contrast, the intraspecific studies of Sabat et al. [72] with a generalist species, the rufous-collared sparrow (*Zonotrichia capensis*), showed that sparrows that consumed food at a lower trophic level had higher BMR compared to birds that used more animal food. In that case, the higher BMR might be due to detoxification pathway activated by secondary compounds in plants [78], [79]. Our results showed slightly lower relative BMR in granivores compared to insectivores, which might be due to the size of the birds, because granivores were slightly bigger than insectivores in this study. However, also the detoxification capacity was lower in granivores, which means that the secondary compounds of the plants most probably do not increase the BMR in this case. Since the number of species was relatively small in our study further investigation is still needed to confirm the relation between metabolic rate, diet and detoxification capacity.

To conclude, the study species differ from each other in their hepatic EROD activity and the difference is related to their feeding habits and migratory status. Migratory species likely use more variable food with higher amounts of secondary compounds both in their breeding and overwintering habitats and have therefore developed higher detoxification capacity, which makes them better adapted to natural and man-made toxic compounds than non-migratory species. The lower EROD activity in migratory granivores compared to insectivores may be related to their narrower and less natural toxins containing diet, which means that their MFO system may not have evolved as effectively as in insectivores. Higher metabolic activity and oxygen consumption can increase ROS production and probably affect their detoxification ability as well. Nonetheless the metabolic rate did not seem to be the main reason for the variation in species detoxification capacity in this study, because there was no correlation between relative BMR and EROD activity even when the phylogeny was taken into account. EROD activity instead correlated positively with relative liver mass, suggesting that the detoxification capacity is related to organ size. However, the number of species as well as the sample size within the species was relative small, so a critical view is needed when drawing conclusions from the results. Our study shows that there is a lot of species-specific variation in basal EROD activity, which means that direct inter-specific comparison of pollution induced EROD activities cannot be done without first knowing the basal level of EROD activity in each species. Also, the EROD activity measurements at different times of the year would be valuable to see whether EROD activity shows seasonal variation in birds. Moreover, in further studies it would be important to measure both granivorous and insectivorous migrants from overwintering areas to see whether the diet in overwintering habitat affects to the variation in EROD activity. The knowledge of basal levels of EROD activities among the species is important to improve its use as biomarker in ecotoxicological studies. The increasing knowledge of tolerance and detoxification ability will also help in identifying the species most sensitive to environmental pollution and in understanding the mechanisms that may decrease bird populations in contaminated areas. Our results suggest that granivores are likely more vulnerable to environmental contaminants than insectivores and migratory birds are likely less susceptible to contamination load than resident birds. Diet and migration status however are strongly related to each other, with diet varying a lot between breeding and overwintering habitats.

## Supporting Information

Table S1 **The DNA sequences for the species collected from GenBank.** In some cases a closely related species had to be used as there were no sequences available for species used in this study. To allow the estimation of the length of the basal branches, two outgroups (*Corvus* and *Oriolus*) were included.
(DOC)
