# Supplementary material for: Variation of Basal EROD Activities in Ten Passerine Bird Species – Relationships with Diet and Migration Status
Source: PLoS One. 2012 Mar 29;7(3):e33926. doi: 10.1371/journal.pone.0033926 (PMC3315499; doi:10.1371/journal.pone.0033926)
Supplement: Table S1 — The DNA sequences for the species collected from GenBank. In some cases a closely related species had to be used as there were no sequences available for species used in this study. To allow the estimation of the length of the basal branches, two outgroups (Corvus and Oriolus) were included. (DOC) [file pone.0033926.s001.doc]

**Table S1.**

| **Gene** | **Accession number** | **Species** | **Notes** |
| --- | --- | --- | --- |
|  |  |  |  |
| cytb | AY156270 | Corvus corax | outgroup |
| cytb | FJ821132 | Oriolus flavocinctus | outgroup |
| cytb | AJ004244 | Acrocephalus schoenobaenus |  |
| cytb | AJ004303 | Acrocephalus scirpaceus |  |
| cytb | AY495384 | Carduelis chloris |  |
| cytb | DQ474043 | Cyanistes caeruleus |  |
| cytb | EF529944 | Emberiza schoeniclus |  |
| cytb | AY495389 | Fringilla coelebs |  |
| cytb | GU460237 | Hirundo rustica |  |
| cytb | AY495412 | Parus major |  |
| cytb | AY495393 | Passer domesticus |  |
| cytb | AJ004326 | Phylloscopus trochilus |  |
|  |  |  |  |
| c-MYC | EF568191 | Corvus corax | outgroup |
| c-MYC | AF377276 | Oriolus oriolus | outgroup |
| c-MYC | EF568224 | Acrocephalus dumetorum | in place of Acrocephalus scirpaceus |
| c-MYC | EF568247 | Carduelis carduelis | in place of Carduelis chloris |
| c-MYC | EF568248 | Emberiza citrinella | in place of Emberiza schoeniclus |
| c-MYC | EF568244 | Fringilla coelebs |  |
| c-MYC | AF377270 | Hirundo rustica |  |
| c-MYC | EF568215 | Parus caeruleus |  |
| c-MYC | EF568214 | Parus major |  |
| c-MYC | EF568232 | Passer domesticus |  |
|  |  |  |  |
| myoglobin | FJ357980 | Corvus corone | outgroup |
| myoglobin | AY228329 | Oriolus oriolus | outgroup |
| myoglobin | FJ883107 | Acrocephalus schoenobaenus |  |
| myoglobin | FJ883111 | Acrocephalus scirpaceus |  |
| myoglobin | EU878695 | Carduelis chloris |  |
| myoglobin | EU878706 | Fringilla coelebs |  |
| myoglobin | AY064258 | Hirundo rustica |  |
| myoglobin | AY228310 | Parus major |  |
| myoglobin | EF449710 | Passer domesticus |  |
| myoglobin | AY228311 | Passer montanus |  |
| myoglobin | DQ673888 | Phylloscopus trochilus |  |
|  |  |  |  |
| RAG2 | AY443132 | Corvus corone | outgroup |
| RAG2 | AY443184 | Oriolus larvatus | outgroup |
| RAG2 | EF568297 | Carduelis carduelis | in place of Carduelis chloris |
| RAG2 | AY443143 | Emberiza schoeniclus |  |
| RAG2 | AY443148 | Fringilla montifringilla | in place of Fringilla coelebs |
| RAG2 | AY443155 | Hirundo rustica |  |
| RAG2 | AY443197 | Parus major |  |
| RAG2 | EF568287 | Passer domesticus |  |
| RAG2 | AY799844 | Phylloscopus collybita |  |
|  |  |  |  |
| ZENK3UTR | EF568123 | Corvus corax | outgroup |
| ZENK3UTR | EF568129 | Oriolus chinensis | outgroup |
| ZENK3UTR | EF568160 | Acrocephalus dumetorum | in place of Acrocephalus scirpaceus |
| ZENK3UTR | EF568182 | Carduelis carduelis | in place of Carduelis chloris |
| ZENK3UTR | EF568183 | Emberiza citrinella | in place of Emberiza schoeniclus |
| ZENK3UTR | EF568179 | Fringilla coelebs |  |
| ZENK3UTR | EF568149 | Parus caeruleus |  |
| ZENK3UTR | EF568148 | Parus major |  |
| ZENK3UTR | EF568168 | Passer domesticus |  |
| ZENK3UTR | EF568158 | Phylloscopus trochilus |  |
|  |  |  |  |
| ZENK | EF568305 | Corvus corax | outgroup |
| ZENK | EF568311 | Oriolus chinensis | outgroup |
| ZENK | EF568343 | Acrocephalus dumetorum | in place of Acrocephalus scirpaceus |
| ZENK | EF568366 | Carduelis carduelis | in place of Carduelis chloris |
| ZENK | EF568367 | Emberiza citrinella | in place of Emberiza schoeniclus |
| ZENK | EF568363 | Fringilla coelebs |  |
| ZENK | EF568332 | Parus caeruleus |  |
| ZENK | EF568331 | Parus major |  |
| ZENK | EF568351 | Passer domesticus |  |
| ZENK | EF568341 | Phylloscopus trochilus |  |
